# Blocking glycine receptors reduces neuroinflammation and restores neurotransmission in cerebellum through ADAM17-TNFR1-NF-κβ pathway

**DOI:** 10.1186/s12974-020-01941-y

**Published:** 2020-09-11

**Authors:** Yaiza M. Arenas, Andrea Cabrera-Pastor, Nora Juciute, Eloy Mora-Navarro, Vicente Felipo

**Affiliations:** 1 Laboratory of Neurobiology, Príncipe Felipe Research Center Valencia, C/Eduardo Primo Yúfera 3, 46012 Valencia, Spain; 2 Laboratory of Neurological Impairment, Health Research Institute INCLIVA, 46010 Valencia, Spain

**Keywords:** Hyperammonemia, Glycine receptor, ADAM17, TNFR1, Purkinje neuron, Neuroinflammation

## Abstract

**Background:**

Chronic hyperammonemia induces neuroinflammation in cerebellum, with glial activation and enhanced activation of the TNFR1-NF-kB-glutaminase-glutamate-GABA pathway. Hyperammonemia also increases glycinergic neurotransmission. These alterations contribute to cognitive and motor impairment. Activation of glycine receptors is reduced by extracellular cGMP, which levels are reduced in cerebellum of hyperammonemic rats in vivo.

We hypothesized that enhanced glycinergic neurotransmission in hyperammonemic rats (1) contributes to induce neuroinflammation and glutamatergic and GABAergic neurotransmission alterations; (2) is a consequence of the reduced extracellular cGMP levels. The aims were to assess, in cerebellum of hyperammonemic rats, (a) whether blocking glycine receptors with the antagonist strychnine reduces neuroinflammation; (b) the cellular localization of glycine receptor; (c) the effects of blocking glycine receptors on the TNFR1-NF-kB-glutaminase-glutamate-GABA pathway and microglia activation; (d) whether adding extracellular cGMP reproduces the effects of strychnine.

**Methods:**

We analyzed in freshly isolated cerebellar slices from control or hyperammonemic rats the effects of strychnine on activation of microglia and astrocytes, the content of TNFa and IL1b, the surface expression of ADAM17, TNFR1 and transporters, the phosphorylation levels of ERK, p38 and ADAM17. The cellular localization of glycine receptor was assessed by immunofluorescence. We analyzed the content of TNFa, IL1b, HMGB1, glutaminase, and the level of TNF-a mRNA and NF-κB in Purkinje neurons. Extracellular concentrations of glutamate and GABA were performed by in vivo microdialysis in cerebellum. We tested whether extracellular cGMP reproduces the effects of strychnine in ex vivo cerebellar slices.

**Results:**

Glycine receptors are expressed mainly in Purkinje cells. In hyperammonemic rats, enhanced glycinergic neurotransmission leads to reduced membrane expression of ADAM17, resulting in increased surface expression and activation of TNFR1 and of the associated NF-kB pathway. This increases the expression in Purkinje neurons of TNFa, IL-1b, HMGB1, and glutaminase. Increased glutaminase activity leads to increased extracellular glutamate, which increases extracellular GABA. Increased extracellular glutamate and HMGB1 potentiate microglial activation. Blocking glycine receptors with strychnine or extracellular cGMP completely prevents the above pathway in hyperammonemic rats.

**Conclusions:**

Glycinergic neurotransmission modulates neuroinflammation. Enhanced glycinergic neurotransmission in hyperammonemia would be due to reduced extracellular cGMP. These results shed some light on possible new therapeutic target pathways for pathologies associated to neuroinflammation.

## Background

Rats with chronic hyperammonemia reproduce the cognitive impairment and motor in-coordination shown by cirrhotic patients with minimal hepatic encephalopathy and are a good model to identify the underlying mechanisms and to test treatments to improve them. Chronic hyperammonemia induces neuroinflammation which alters glutamatergic and GABAergic neurotransmission in cerebellum and hippocampus leading to cognitive and motor impairment [1–8].

Hyperammonemic rats show neuroinflammation in cerebellum, with microglia and astrocytes activation and increased content of TNFa (tumor necrosis factor alpha) and membrane expression of the TNFα receptor 1 (TNFR1). This is associated with increased nuclear factor kappa B (NF-κB), leading to increased glutaminase levels, which increases glutamate formation and its extracellular concentration. The increased transport of glutamate by activated astrocytes is associated with increased amount and reversal of the function of the GABA transporter type 3 (GAT-3) resulting in increased extracellular γ-aminobutyric acid (GABA) levels, which induce motor incoordination and impairs the ability to learn a conditional discrimination task in the Y maze [3, 5, 6, 9].

Hyperammonemia also alters glycinergic neurotransmission in the cerebellum in vivo and these alterations contribute to the mechanisms that impair learning in the Y maze in hyperammonemia [10]. As far as we know the effects of glycinergic neurotransmission on neuroinflammation have not been studied.

It has been reported that, in cultured adipocytes, glycine strongly stimulates NF-kB activation and that this effect is completely prevented by blocking the glycine receptor using the antagonist strychnine. In contrast, glycine inhibits the activation of NF-kB induced by TNFa in these adipocytes [11].

It is likely that the altered glycinergic neurotransmission in hyperammonemic rats may be involved in the induction of neuroinflammation and in the mechanisms by which it alters glutamatergic and GABAergic neurotransmission in the cerebellum.

This idea is supported by the fact that extracellular cyclic guanosine monophosphate (cGMP), at physiological (nM) concentrations, modulates glycine receptors, reducing its activation [12]. In hyperammonemia, the levels of extracellular cGMP are reduced in cerebellum in vivo [5, 13–15]. Chronic intracerebral administration of extracellular cGMP to rats with chronic hyperammonemia reduces neuroinflammation, including microglia and astrocytes activation and membrane expression of the TNFα receptor 1 (TNFR1). This is associated with reduced nuclear NF-κB, glutaminase content and extracellular glutamate, reduced amount of the GABA transporter GAT-3 in activated astrocytes, reduced extracellular GABA in cerebellum and restoration of motor coordination [3].

However, it has not been studied whether the above effects of extracellular cGMP in cerebellum of hyperammonemic rats could be mediated by the inhibition of glycine receptors.

Based on the above data, we propose the hypotheses that in chronic hyperammonemia increased activation of glycine receptors may contribute to neuroinflammation, including activation of microglia and astrocytes, and to increased membrane expression of TNFR1 and activation of the associated signal transduction pathway, leading to increased levels of extracellular glutamate and GABA. The reduced concentration of extracellular cGMP would contribute to the increased activation of glycinergic neurotransmission and the subsequent effects. If this is the case, adding extracellular cGMP to cerebellar slices ex vivo should reverse the effects of increased glycinergic neurotransmission, mimicking the effects of the antagonist strychnine.

The aims of this work were to assess, in cerebellar slices of hyperammonemic rats, (1) whether blocking the glycine receptor with the antagonist strychnine reduces neuroinflammation in cerebellum; (2) the cellular localization of glycine receptor; (3) the effects of blocking glycine receptors on the TNFR1-NF-kB-glutaminase-glutamate-GABA pathway and microglia activation; (4) whether addition of extracellular cGMP to cerebellar slices reproduces the effects of strychnine.

## Methods

### Rats

Male Wistar rats were made hyperammonemic by feeding them an ammonium-containing diet as previously described [16]. The experiments were approved by the Comite de Experimentación y Bienestar Animal (CEBA) of our Center and by the Conselleria de Agricultura of Generalitat Valenciana and were performed in accordance with guidelines of the Directive of the European Commission (2010/63/EU) for care and management of experimental animals.

### Analysis of protein content and phosphorylation in cerebellar slices by western blot

Control and hyperammonemic rats were sacrificed at 4 weeks of hyperammonemia and the cerebelli were immediately immersed into ice-cold Krebs buffer (in mmol/L): NaCl 119, KCl 2.5, KH_2_PO_4_ 1, NaHCO_3_ 26.2, CaCl_2_ 2.5, and glucose 11, aerated with 95% O_2_ and 5% CO_2_ at pH 7.4. Cerebellar slices (400 μm-thick, transversal) were cut and incubated for 20 min at 35.5 °C in Krebs buffer for stabilization. Seventy-five micrometer strychnine to block the glycine receptor, 1 μM bisindolyl for PKC inhibition, and the treatment with 40 nM cGMP were added to slices and incubated for 30 min. Slices were collected and homogenized by sonication for 20 s in a buffer (Tris-HCl 66 mM pH 7.4, SDS 1%, EGTA 1 mM, glycerol 10%, leupeptin 0.2 mg/mL, NaF 1 mM, Na orto-vanadate 1 mM) for analysis of protein content and phosphorylation by western blot. Samples were subjected to immunoblotting as in Felipo et al. [17], using antibodies against Interleukin 1 beta (IL-1β) (1:500) from R&D Systems; ERK (1:1000) and pERK phosphorylated at Tyr204 (1:1000) from Sta Cruz, p38 (1:1000) and p38 phosphorylated at Thr180/Tyr182 (1:500) from cell signaling; ADAM metallopeptidase domain 17 (ADAM17; 1:250); and ADAM17 phosphorylated at Thr735 (1:2000) from Abcam and TNFα (1:1000) from R&D Systems. Phosphorylation levels were normalized to the total amount of the respective proteins. As a control for protein loading, the same membranes used to quantify the amount of proteins were incubated with an antibody against Actin (1:5000) from Abcam or GADPH (1:10000) from Millipore depending on the molecular mass of the other proteins. Secondary antibodies were anti-rabbit (cat# A8025), anti-goat (cat# A7650), or anti-mouse (cat# A3562) IgG, 1:4000 dilution conjugated with alkaline phosphatase from Sigma (St. Louis, MO). The images were captured using the ScanJet 5300C (Hewlett-Packard, Amsterdam, Netherlands), and band intensities quantified using the Alpha Imager 2200, version 3.1.2 (AlphaInnotech Corporation, San Francisco).

### Analysis of membrane expression of ADAM17 and of receptors or transporters

Membrane expression of ADAM17, TNFR1, glutamate aspartate transporter 1 (GLAST), glutamate transporter 1 (GLT1), and GAT3 in cerebellar slices was analyzed by cross-linking with BS3 (bis(sulfosuccinimidyl) suberate, Pierce cat# 21580, Rockford, IL) and using antibodies against ADAM17 (1:250) from Abcam, TNFR1 (1:250) from Abcam, GLAST (1:4000) from Novus, GLT1 (1:1000) from Thermo PAS, and GAT-3(1:500) from Alomone LABS. After the treatments (see above), slices were added to tubes containing ice-cold Krebs buffer with or without 2 mM BS3 and incubated for 30 min at 4 °C with gentle shacking. Cross-linking was terminated by quenching the reaction with 100 mM glycine (10 min, 4 °C). The slices were homogenized by sonication for 20 s. Samples treated or not with BS3 were analyzed by western blot as described above. The surface expression of each subunit was calculated as the difference between the intensity of the bands without BS3 (total protein) and with BS3 (non-membrane protein) as described by [18].

### Immunohistochemistry

After the incubation of cerebellar slices with different treatments, as described above, slices were fixed in 4% paraformaldehyde in 0.1 M phosphate buffer (pH 7.4) during 24 h at 4 °C. Paraffin-embedded slices were cut and mounted on a coated slide glass, processed with the Envision Flex+kit (DAKO) blocking endogenous peroxidase activity for 5 min and incubated with antibodies against Iba1 (Wako; 1:300 for 30 min), GFAP (Dako; ready to use for 20 min), Glutaminase I (Novus; 1:100 for 60 min), TNFα (Abcam; 1:200 for 45 min), IL1β (Abcam; 1:400 for 60 min), or high mobility group box 1 protein (HMGB1) (Abcam; 1:400 for 60 min). The reaction was visualized by Envision Flex+horseradish peroxidase for 20 min and finally diaminobenzidine for 10 min. Sections were counterstained with Mayer’s hematoxylin for 5 min.

### Analysis of astrocytes and microglia activation

Analysis of ionized calcium-binding adapter molecule 1 (Iba1) and glial fibrillary acidic protein (GFAP) staining was performed in the white matter of cerebellar slices using the Image J software. Cerebellar slices from six animals per group were used. Microglial activation was assessed by measuring the area and perimeter of Iba1-stained cells in 10 randomly selected 56× fields per section. The results were expressed in square micrometers and micrometers, respectively. For GFAP quantification the area of interest was selected. Using auto local threshold and analyze particle functions the intensity thresholds and size filter were applied. To measure the total amount of GFAP, no size filter was applied. For each rat at least 10 56× fields were quantified. The result was expressed as percentage of area stained by GFAP.

### Analysis of glutaminase I, TNFα, IL1β, and HGMB1 content in Purkinje neurons

Analysis of staining of each protein was performed in Purkinje neurons using the Image J software. Purkinje neurons were manually selected using a freehand selection of ROI manager function and the mean intensity (M.I.) of staining for glutaminase I, TNFα, IL1β, and HGMB1 were recorded. The analysis was performed on at least 10 40× fields for each rat.

### Immunofluorescence analysis of glycine receptor and of NF-κB p50

Double immunofluorescences were performed to assess glycine receptor co-localization with microglia (using Iba1, 1:300, Abcam), astrocytes (using GFAP, Sigma, 1:400), and Purkinje neurons (using Calbindin, 1:200, Abcam).

Analysis of p50 subunit of NF-κB was performed by immunofluorescence. Cerebellar slices from six different animals per group were selected, washed in 0.1 M phosphate buffer, and blocked with normal serum from the same species as the secondary antibody before being incubated overnight with primary antibody (NF-κB p50, 1:200; Iba1, 1:300) from Abcam diluted in blocking buffer and secondary fluorescent antibody (1:400) from Invitrogen. The nuclei were counterstained with DAPI (Sigma-Aldrich) and sections cover slipped. The images were observed under a confocal microscope (Leica TCS-SP2-AOBS) and photographically recorded.

Nuclear and cytoplasmic intensity of p50 subunit was analyzed using ImageJ (1.48v). Nuclei were outlined using the ROI manager function on DAPI blue channel and the selection was applied on a green channel (p50) to measure fluorescence. Mean intensity (M.I.) for each nucleus was measured. For cytoplasmic analysis of p50 subunit of NF-κB, green channels were used and cytosol of each cell was manually outlined using a freehand selection of ImageJ and mean intensity (MI) recorded. Results are expressed as nuclear/cytoplasmic ratio of p50 subunit of NF-κB.

Fluorescence in situ hybridization was performed to detect TNFα mRNA expression in cerebellar slices after the treatments (see above) as in [19]. In brief, slices were deparaffined and rehydrated and tissue was digested with proteinase K (Ambion-Life Technologies). A fluorescein-conjugated probe of 23 nucleotides (50 μM; Exiqon) was diluted in hybridization solution (50 ng/μl) with 30% formamide and denatured at 80 °C for 2 min. The slices were incubated for 16 h in a humidified hybridization chamber at 60 °C. The next day, two stringency washes were performed with 1X SSC at 48 °C for 15 min and 1X SSC at room temperature for 15 min. The slices were counterstained with 4′,6-diamidino-2-phenylindole (DAPI; Sigma; 5 μg/ml). The slices were observed under a confocal microscope and photographically recorded. To quantify the content of TNFα mRNA in Purkinje neurons, cells were manually outlined using ImageJ and mean intensity (M.I.) was measured. The ROI manager function was used to quantify the fluorescence intensity of the probe. Cells were selected manually and the value of total TNFα mRNA content was obtained using the “Mean Gray Value” parameter. The value of this parameter is directly proportional to the intensity of the mRNA expression. The results were expressed as a grayscale mean.

### Analysis of extracellular GABA and glutamate in cerebellum by in vivo microdialysis

Control and hyperammonemic rats were anesthetized with isoflurane at 5% for induction and 1.5–3% for maintenance. A microdialysis guide was implanted in the cerebellum (AP-10.2, ML-1.6, and DV-1.2), as in Cabrera-Pastor et al. [13]. After 48 h, a microdialysis probe was implanted in the freely moving rat. Probes were perfused (3 μL/min) with artificial cerebrospinal fluid (in mM): NaCl, 145; KCl, 3.0; CaCl_2_, 2.26; buffered at pH 7.4 with 2 mM sodium phosphate. After a 2–3 h stabilization period, samples were collected every 30 min. When indicated, strychnine (4 μM) was administered through the microdialysis probe to block glycine receptor. All samples were stored at 80 °C until analysis of extracellular GABA and glutamate. GABA and glutamate were analyzed by high-performance liquid chromatography (HPLC) after derivatization with o-phtalaldehyde and fluorescence detection with excitation at 340 nm and emission at 460 nm as previously described [20].

### Statistical analysis

Results are expressed as mean ± standard error. All statistical analyses were performed using the software program GraphPad Prism. The number of samples necessary to analyze the content or phosphorylation of different proteins by western blot was very different and was adjusted in each case to reduce variability and to obtain reliable results. The variability was due to different causes: abundance of the protein analyzed, quality of the antibody used, or magnitude of the changes induced by hyperammonemia or by treatments. Normality distribution was assessed using D’Agostino and Pearson omnibus test and Shapiro-Wilk normality tests. Differences in variances of normally distributed data were assessed using Bartlett’s test. Data with the same variance across groups were analyzed by a parametric one-way analysis of variance (ANOVA) followed by Turkey post hoc test when more than two groups. Data with different variance across groups were analyzed using the non-parametric Kruskal-Wallis test followed by Dunnett’s post hoc test. A confidence level of 95% was accepted as significant. The number of rats used for each parameter and statistical procedure used in each case is indicated in the corresponding figure legend.

## Results

### Blocking glycine receptor reduces astrocytes and microglia activation in the cerebellum of hyperammonemic rats

It has been shown that hyperammonemia enhances glycinergic neurotransmission in the cerebellum in vivo [10]. We assessed whether reducing glycinergic neurotransmission by treatment with strychnine, an antagonist of glycine receptor, reduces microglial, and/or astrocytes activation and neuroinflammation in freshly isolated cerebellar slices from hyperammonemic rats.

**Fig. 1:**
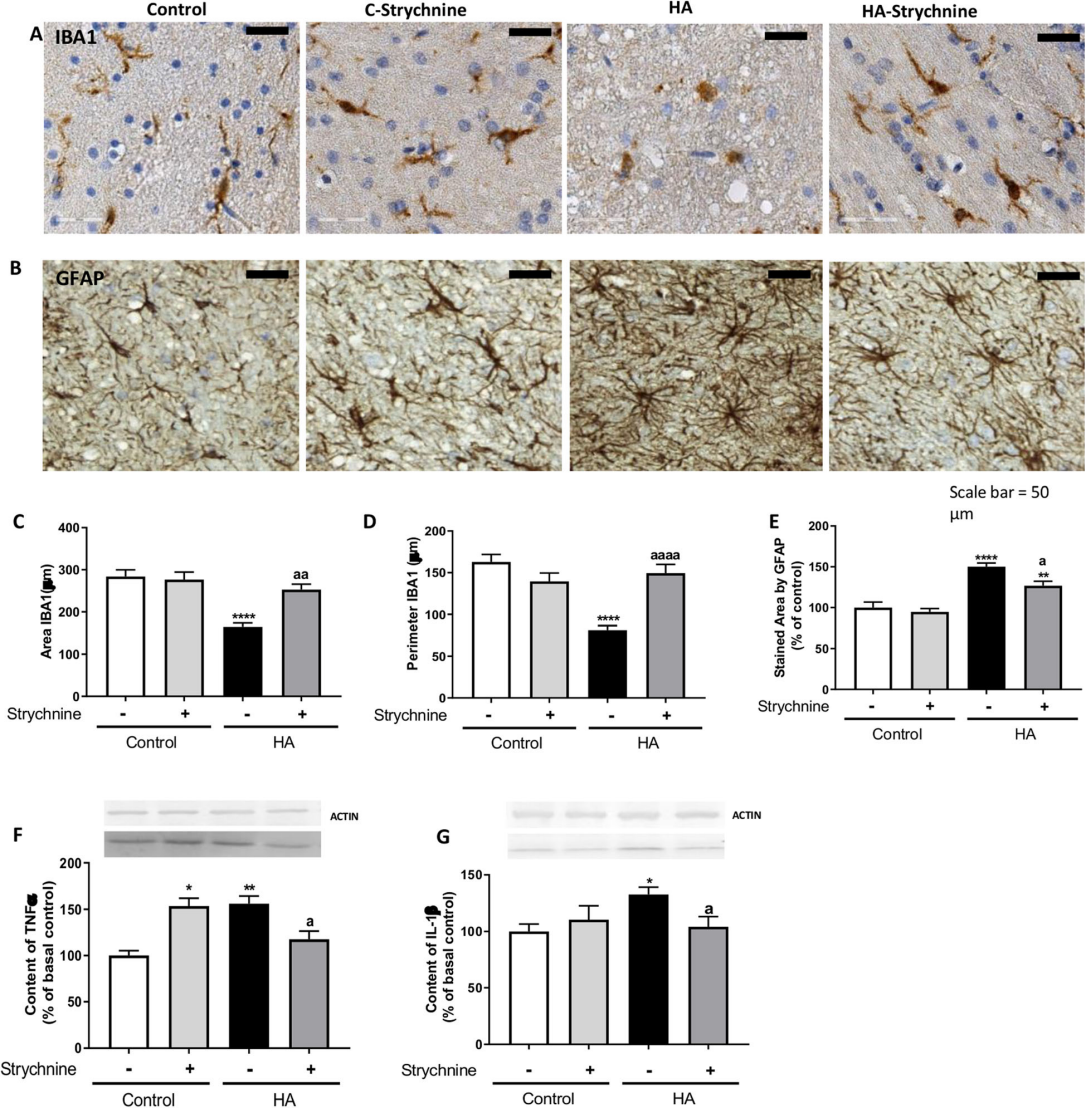
Blocking glycine receptor with strychnine reduces activation of microglia and astrocytes and normalizes the content in TNFα and IL1β in cerebellum from hyperammonemic (HA) rats. Immunohistochemistry was performed in cerebellar slices from control and hyperammonemic rats treated or not with strychnine as indicated in methods with DAB staining using antibodies against IBA1 (**a**) and GFAP (**b**). Hyperammonemic rats show altered morphology of microglia and astrocytes stained indicating activation. Treatment with strychnine reduces activation. To analyze the activation of microglia the area (**c**) and perimeter (**d**) of the cells was measured using the Image J analysis software as described in the “Methods” section. The GFAP stained area was measured as an indicator of astrocytes activation (**e**) as described in methods. The content of TNFα (**f**) and IL1β (**g**) was measured by western blot. Values are the mean ± SEM of 6 rats per group in **a-e** and of 13–30 rats per group in **f-g**. Values significantly different from control rats are indicated by asterisk and from hyperammonemic rats are indicated by “a”. *p < 0.05, **p < 0.01, ****p < 0.0001; a *p* < 0.05, aa *p* < 0.01, aaaa *p* < 0.0001. Data were analyzed using a one-way analysis of variance (ANOVA) followed by Turkey post hoc

Microglia were activated in cerebellar slices of hyperammonemic rats, as reflected by changes in morphology to a less ramified form (Fig. 1a). Strychnine normalized the morphology of microglia (Fig. 1a), indicating reduction of microglial activation.

The activation of microglia was quantified by measuring its area and perimeter, which are reduced in activated microglia. In white matter, the area stained by Iba-1 was reduced in hyperammonemic rats to 165 ± 9μm^2^ (*p* < 0.0001) compared to 284 ± 16μm^2^ in control rats. Treatment with strychnine normalized (*p* < 0.01) the area stained by Iba-1 in hyperammonemic rats to 253 ± 13μm^2^ (Fig. 1c).

The perimeter of microglia was reduced (*p* < 0.0001) in hyperammonemic rats to 81 ± 5 μm compared to 163 ± 9 μm in control rats. Treatment with strychnine reduced microglia activation in hyperammonemic rats, increasing the perimeter to 150 ± 10 μm (Fig. 1d).

Hyperammonemic rats show also activation of astrocytes in the cerebellum, as reflected by the altered morphology of the astrocytes stained with anti-GFAP (Fig. 1b). Treatment with strychnine normalized astrocytes morphology (Fig. 1b), indicating that the inhibition of glycine receptor reduces activation of astrocytes in hyperammonemic rats.

Astrocyte activation was quantified by analyzing the area stained by GFAP, which was increased in hyperammonemic rats to 150 ± 4% (*p* < 0.0001) of control rats. Treatment with strychnine reduced (*p* < 0.05) the area stained by GFAP in hyperammonemic rats to 127 ± 5% of control rats (Fig. 1e).

### Strychnine normalizes the content of TNFα and IL1β in cerebellar slices of hyperammonemic rats

Activation of microglia and astrocytes is associated with an increase of TNFα and IL1β in cerebellum [3]. We assessed the effect of strychnine on the content of TNFα and IL1β in cerebellar slices. Hyperammonemia increased the content of TNFα to 156 ± 8% (*p* < 0.01) and of IL1β to 132 ± 6% (*p* < 0.05) of control rats. Strychnine normalized the content of both TNFα and IL1β (Fig. 1f and g, respectively).

### Glycine receptor is mainly localized in Purkinje cells

**Fig. 2:**
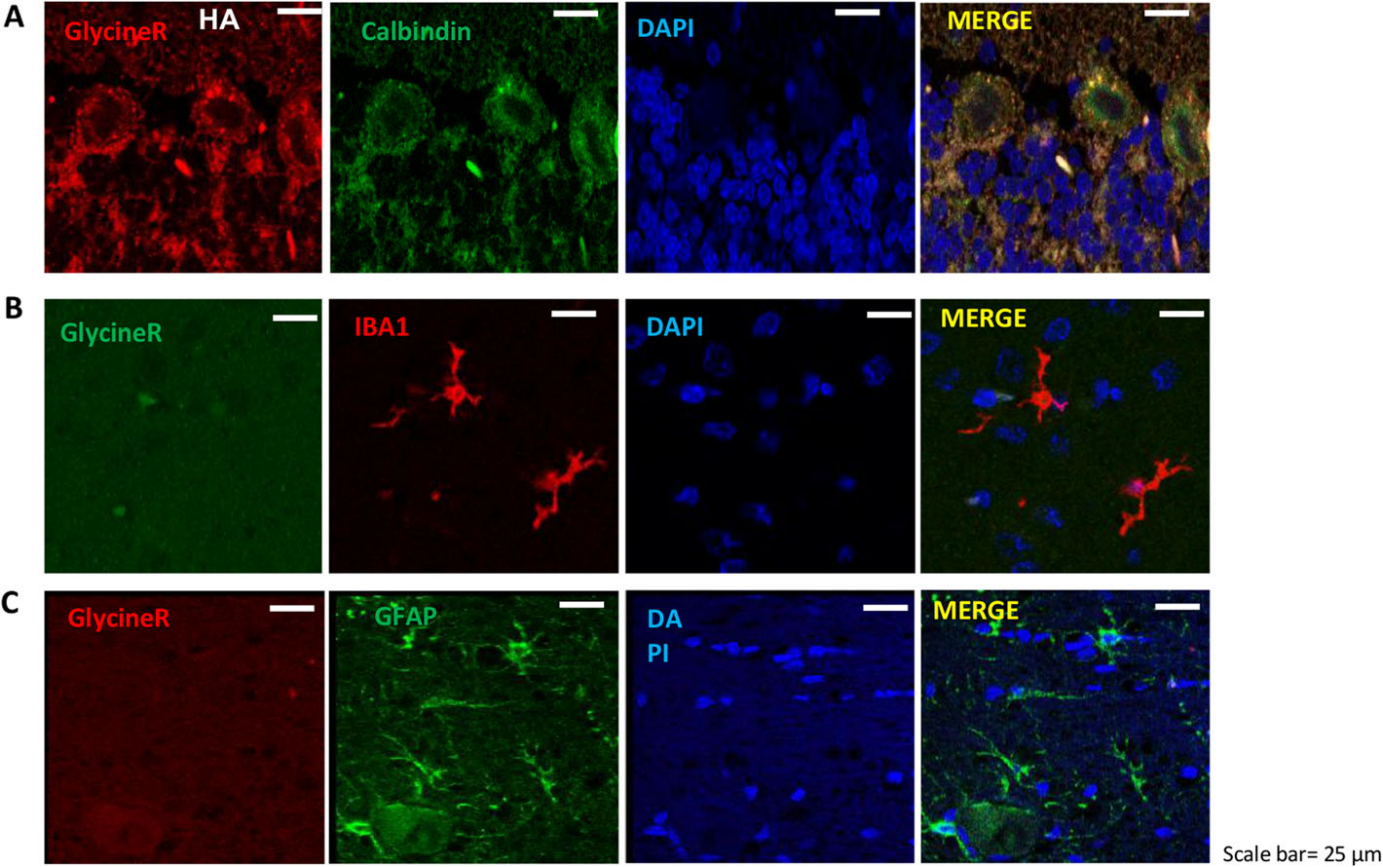
Glycine receptor is mainly localized in Purkinje cells. Double fluorescence staining was performed using anti-glycine receptor with calbindin (**a**), IBA1 (**b**), and GFAP (**c**) as described in methods. Representative images of the double fluorescence staining shown that glycine receptor colocalized with Purkinje cells (**a**) but not with microglia (**b**) or astrocytes (**c**)

The reduction of microglia and astrocytes activation by strychnine could be mediated by glycine receptors located in these glial cells or in neurons. To shed light on this point, we analyzed the localization of glycine receptor in the cerebellum by immunofluorescence. As shown in Fig. 2, double fluorescence staining shows that glycine receptor colocalized with Purkinje cells (Fig. 2a) but not with microglia (Fig. 2b) or astrocytes (Fig. 2c).

These results suggest that in hyperammonemic rats the inhibition of glycine receptor in Purkinje cells would mediate the reduced activation of microglia and astrocytes. We then analyzed the underlying mechanisms.

### Blocking glycine receptor normalizes membrane expression of the TNFα receptor TNFR1 through a PKC and ADAM17-dependent mechanism

It has been reported that increased membrane expression of TNFR1 mediates the induction of neuroinflammation and motor incoordination in hyperammonemia [3].

**Fig. 3:**
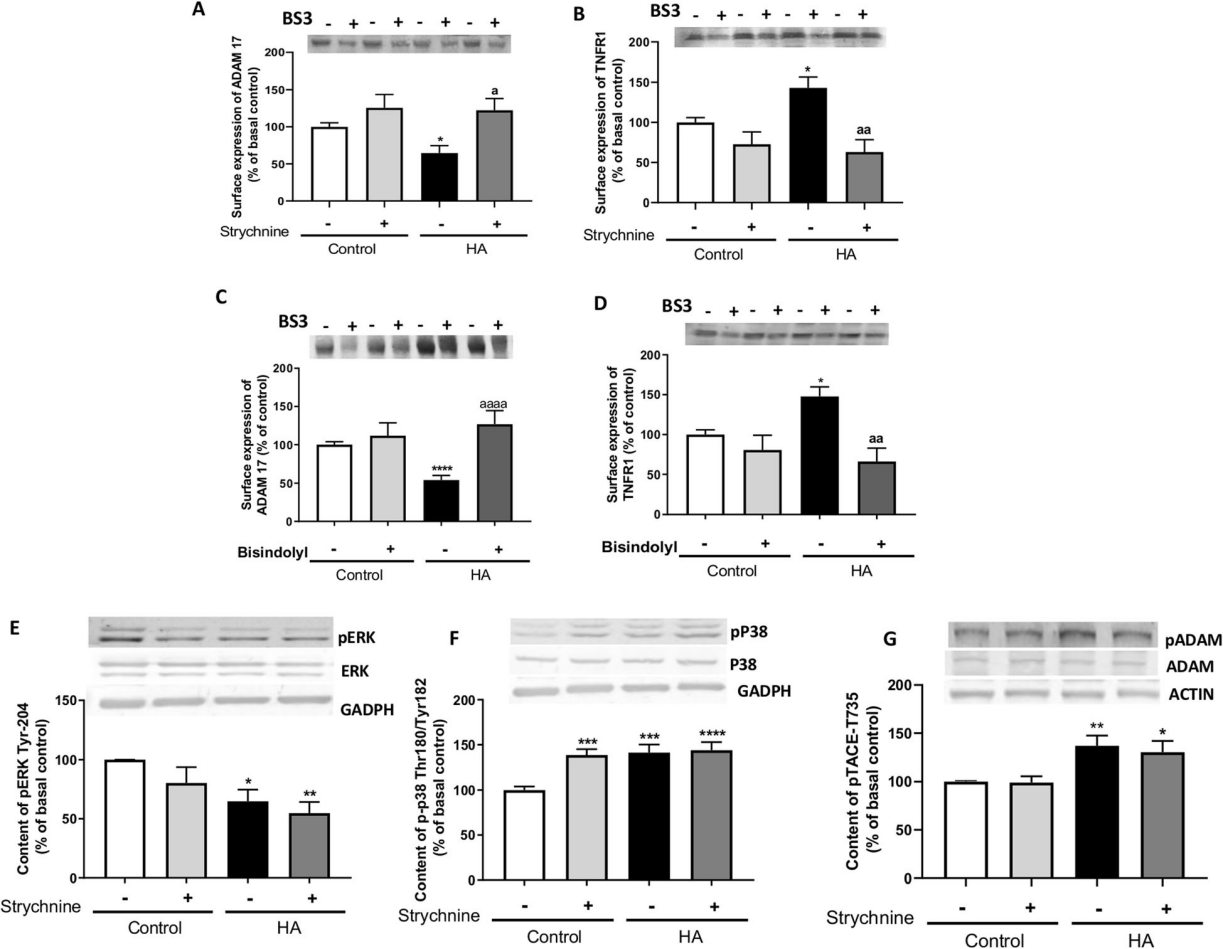
Blocking glycine receptor normalizes membrane expression of the TNFα receptor TNFR1 through a PKC and ADAM17-dependent mechanism. Membrane expression of TNFR1 (**a** and **c**) and ADAM17 (**b** and **d**) were analyzed using BS3 cross-linker procedure as described in the “Methods” section in slices from control and hyperammonemic (HA) rats treated with strychnine (inhibitor of glycine receptor) or bisindolyl (PKC inhibitor). The phosphorylation at Tyr204 of ErK (**e**), at Thr180/Tyr182 of p38 (**f**), and at Thr735 of ADAM17 (**g**) were analyzed by western blot in slices from control and hyperammonemic rats treated or not with strychnine. Phosphorylation levels were normalized to the total amount of the respective proteins (Erk, p38 and ADAM17) and to the control for protein loading (GADPH or actin). Values are the mean ± SEM of 12–41 rats per group. Values significantly different from control rats are indicated by asterisk and from hyperammonemic rats are indicated by “a”. *p < 0.05, **p < 0.01, ****p < 0.0001; a *p* < 0.05, aa *p* < 0.01, aaaa *p* < 0.0001. Data were analyzed using a one-way analysis of variance (ANOVA) followed by Turkey post hoc (in **a**, **c**, **e**, and **f**) and the non-parametric Kruskal-Wallis test followed by Dunnett’s post hoc test (in **b**, **d**, and **g**)

The amount of TNFR1 in the membrane was increased in cerebellar slices of hyperammonemic rats to 143 ± 13% of control rats (Fig. 3a). Treatment with strychnine completely reversed this effect, reducing TNFR1 in the membrane to 63 ± 15% of control rats (Fig. 3a). Membrane expression of TNFR1 may be modulated by shedding mediated by ADAM17 [21, 22]. We therefore assessed the effects of hyperammonemia and of strychnine on membrane expression of ADAM17. As shown in Fig. 3b, the amount of ADAM17 in the membrane is reduced in the cerebellum of hyperammonemic rats to 64 ± 9% of control rats (Fig. 3b). This suggests that hyperammonemia could increase TNFR1 membrane expression by reducing its shedding mediated by ADAM17. Strychnine also completely reversed this effect, increasing ADAM17 in the membrane to 122 ± 16% of control rats (Fig. 3b).

A main mechanism modulating membrane expression of ADAM17 is mediated by PKC [23]. As shown in Fig. 3c and d, bisindolyl, an inhibitor of PKC, normalizes the membrane expression of TNFR1 and ADAM17 to 66 ± 17% and 127 ± 18% of control rats, respectively, reproducing the effects of strychnine treatment.

Membrane expression of ADAM17 may be modulated by phosphorylation in the residue of threonine 735 mediated by the PKC-ERK/p38 pathway [24, 25]. We then assessed whether strychnine modulates membrane expression of ADAM17 through this pathway. Hyperammonemia reduces the phosphorylation at Tyr204 of Erk to 65 ± 9% (Fig. 3e), increases the phosphorylation at Thr180/Tyr182 of p38 to 142 ± 9% (Fig. 3f), and the phosphorylation at Thr735 of ADAM17 to 137 ± 11% (Fig. 3g), of control rats, and these effects are not reversed by strychnine.

These data indicate that reduced membrane expression of ADAM17 in hyperammonemic rats is due to increased PKC activity but is not mediated by the ERK/p38-ADAM17-Thr735 pathway.

### Strychnine normalizes the nuclear content of NF-κB and the levels of glutaminase, TNFα, IL1β, and HMGB1 in Purkinje cells of hyperammonemic rats

Increased membrane expression of TNFR1 in rats with chronic hyperammonemia leads to increased nuclear translocation of p50 subunit of NF-κB and, as a consequence, an increase of TNFα, IL1β, and glutaminase expression [3, 26].

**Fig. 4:**
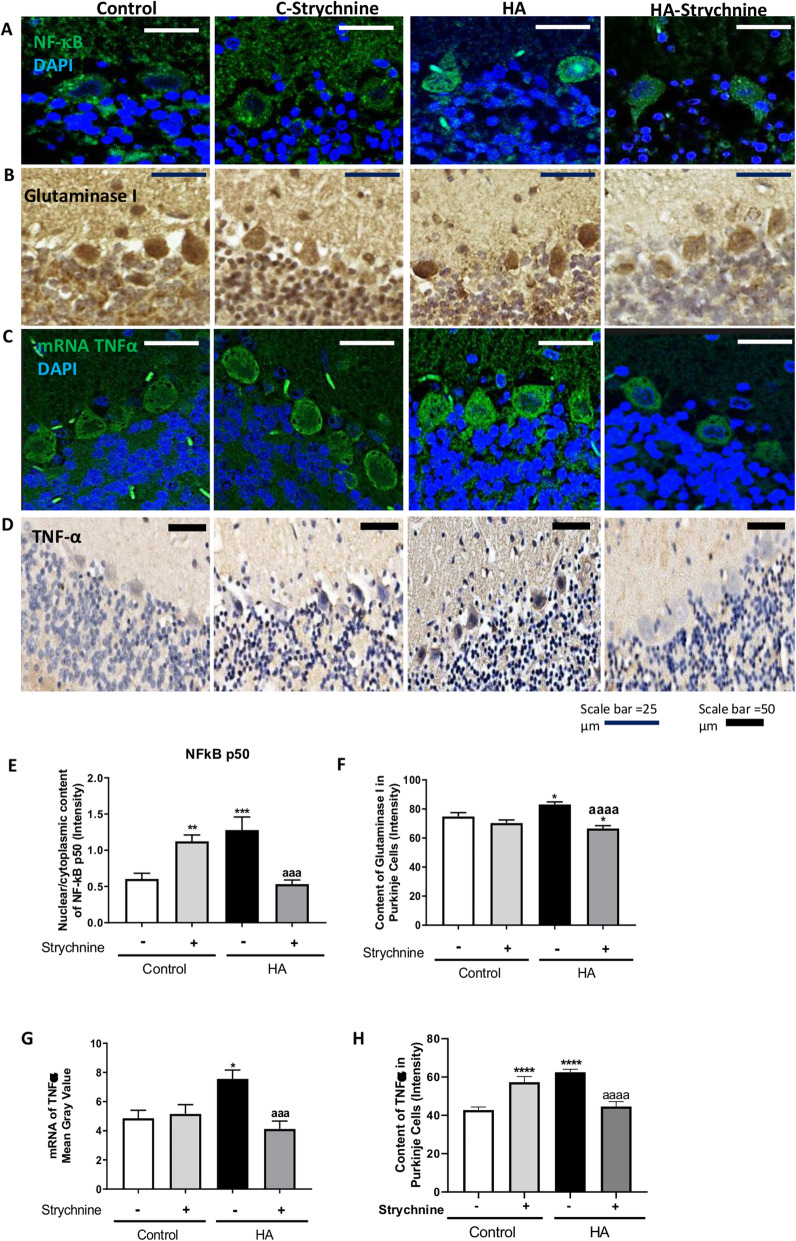
Strychnine normalizes the nuclear content of NF-κB and the levels of glutaminase, TNFα, and mRNA of TNFα in Purkinje cells of hyperammonemic rats. Immunofluorescence was performed using anti-NF-KB (**a**) and anti-mRNA of TNFα (**c**) as described in the “Methods” section. Immunohistochemistry was performed as indicated in methods with DAB staining using antibodies against glutaminase (**b**) and TNFα (**d**). Representative images are shown. The ratio nuclear/cytoplasmic content of NF-KB (**e**), the content of glutaminase (**f**), mRNA of TNFα (**g**), and TNFα (**h**) was quantified in Purkinje cells as described in the “Methods” section. Values are mean ± SEM of 6 rats per group. Values significantly different from control rats are indicated by asterisk and from hyperammonemic rats are indicated by “a”. *p < 0.05, **p < 0.01, ***p < 0.001, ****p < 0.0001; aaa *p* < 0.001, aaaa *p* < 0.0001. Data were analyzed using a one-way analysis of variance (ANOVA) followed by Turkey post hoc

**Fig. 5:**
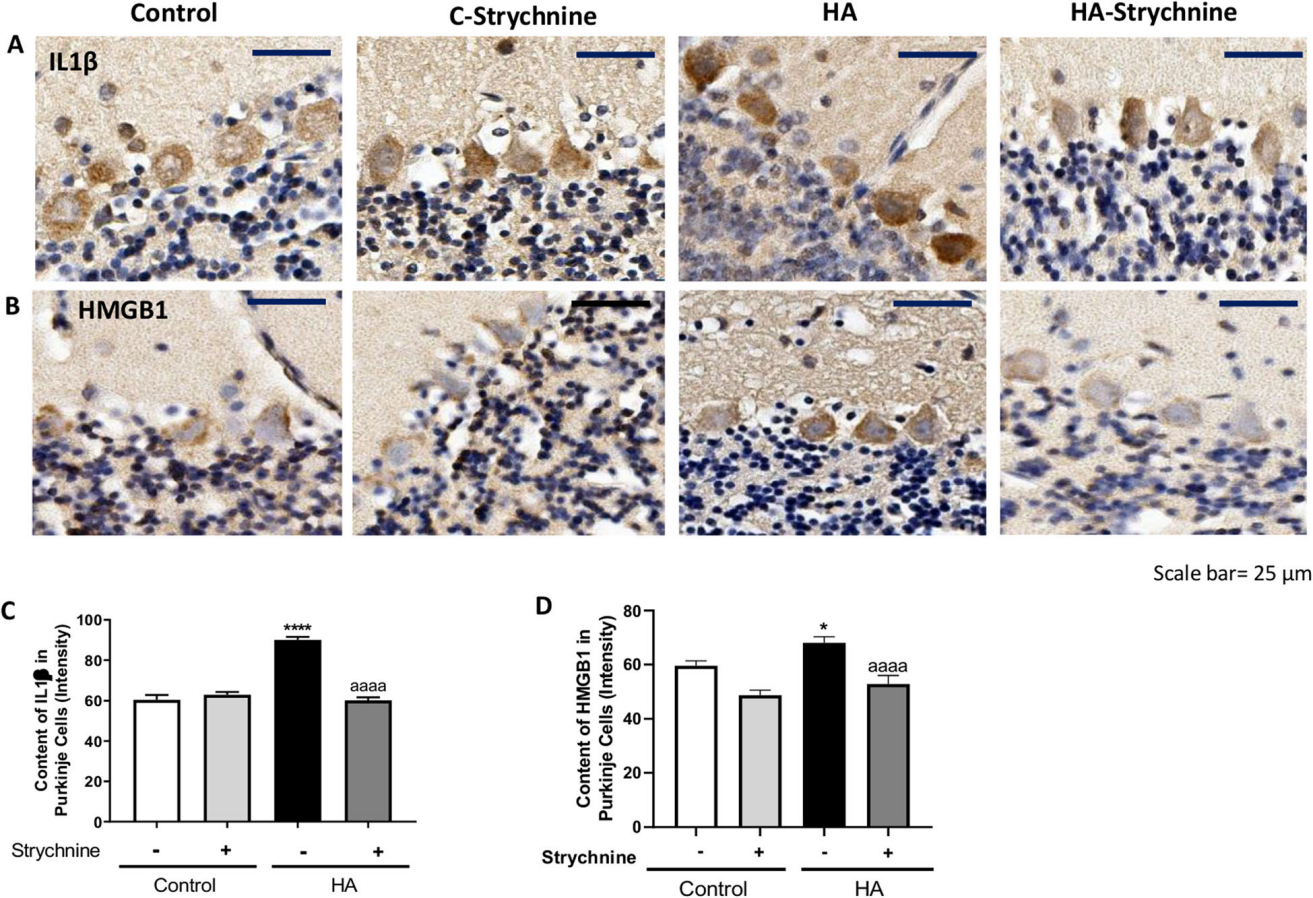
Strychnine normalizes the IL1β and HMGB1 in Purkinje cells of hyperammonemic rats. Immunohistochemistry was performed as indicated in the “Methods” section with DAB staining using antibodies against IL1β (**a**) and HMGB1 (**b**). Representative images are shown. The content of IL1β (**c**) and HMGB1 (**d**) was quantified in Purkinje cells as described in the “Methods” section. Values are mean ± SEM of 6 rats per group. Values significantly different from control rats are indicated by asterisk and from hyperammonemic rats are indicated by “a”. *p < 0.05, ****p < 0.0001; aaaa *p* < 0.0001. Data were analyzed using a one-way analysis of variance (ANOVA) followed by Turkey post hoc

We then assessed whether the reduction of TNFR1 membrane expression by strychnine reduces the NF-κB pathway in cerebellar slices of hyperammonemic rats. As shown in Fig. 4a and e, the nuclear content of the p50 subunit of NF-κB in Purkinje cells is increased (*p* < 0.001) in hyperammonemia and is normalized (*p* < 0.0001) by strychnine. Strychnine also normalized the content of glutaminase (Fig. 4b and f), the amount of mRNA for TNFα (Fig. 4c and g), and the protein content of TNFα (Fig. 4d and h) and IL1β (Fig. 5a and c) in Purkinje cells.

It has been shown that increased nuclear translocation of NF-κB increases HMGB1 expression in neurons which induces microglia activation [27, 28]. In Purkinje neurons, the intensity of HMGB1 staining was higher in hyperammonemic rats than in control rats and strychnine normalized it (Fig. 5b and d). This indicates that strychnine could be reducing the activation of microglia by reducing HMGB1 expression in Purkinje neurons.

### Strychnine reduces extracellular glutamate and GABA concentration and surface expression of GAT3 in the cerebellum of hyperammonemic rats

The increase in glutaminase in hyperammonemia is associated with increased production and extracellular levels of glutamate, increased amount, and reversal of the function of the GABA transporter GAT-3, increased extracellular GABA in the cerebellum and motor in-coordination [3].

**Fig. 6:**
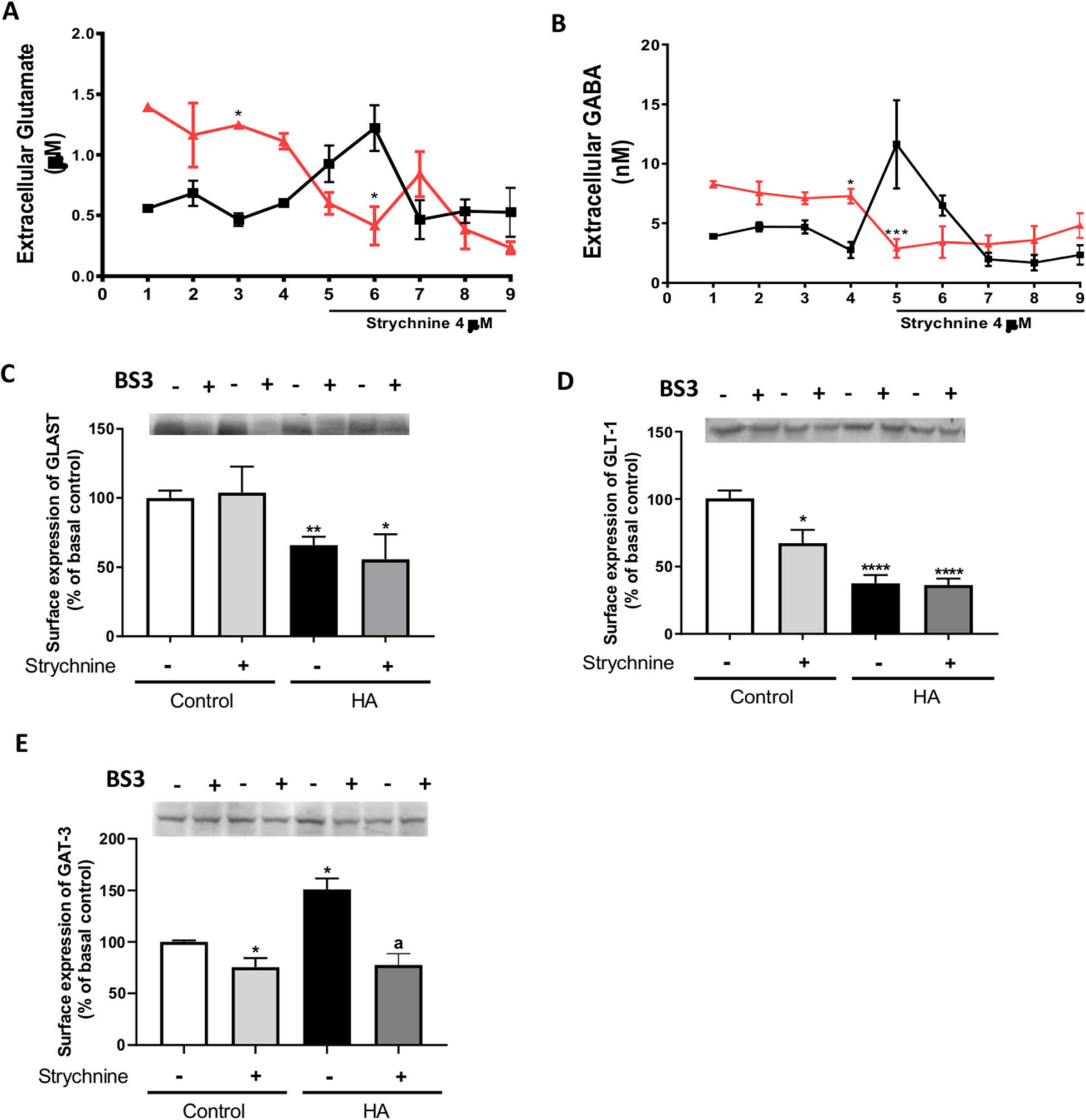
Strychnine reduces extracellular glutamate and GABA concentration and surface expression of GAT3 in the cerebellum of hyperammonemic rats. Extracellular glutamate (**a**) and GABA (**b**) levels were measured in the cerebellum by microdialysis in vivo as indicated in the “Methods” section. Strychnine (4 μM), an inhibitor of glycine receptor, was administered (fraction 5–9) though the microdialysis probe in cerebellum in vivo of control and hyperammonemic rats and extracellular glutamate and GABA were measured as described in the “Methods” section. Surface expression of glutamate transporters, GLAST (**c**) and GLT-1 (**d**), and GABA transporter GAT3 (**e**) were analyzed using BS3 cross-linker procedure as described in the “Methods” section in slices from control and hyperammonemic (HA) rats treated or not with strychnine. Values are mean ± SEM of 7 rats per group in **a-b** and of 8-25 rats per group in **c-e**. Values significantly different from control rats are indicated by asterisk and from hyperammonemic rats are indicated by “a”. *p < 0.05, **p < 0.01, ***p < 0.001, ****p < 0.0001; a *p* < 0.05. Data were analyzed using a one-way analysis of variance (ANOVA) followed by Turkey post hoc (in **a-d**) and the non-parametric Kruskal-Wallis test followed by Dunnett’s post hoc test (in **e**)

We assessed the effects of in vivo treatment with strychnine on extracellular glutamate and GABA by microdialysis in vivo in freely moving rats. Hyperammonemic rats show increased extracellular glutamate and GABA in the cerebellum compared to controls and strychnine administration through the microdialysis probe normalizes extracellular glutamate (Fig. 6a) and GABA (Fig. 6b) in hyperammonemic rats. We then assessed the effects of hyperammonemia and of strychnine on membrane expression of the glutamate transporters GLAST and GLT1 and of the GABA transporter GAT3 in cerebellar slices.

Hyperammonemia reduced the surface expression of GLAST and GLT-1 to 66 ± 6% (*p* < 0.01, Fig. 6c) and 38 ± 6% (*p* < 0.0001, Fig. 6d) of controls, respectively. The surface expression of these transporters is not restored by strychnine.

Hyperammonemia increases the surface expression of GAT3 (151 ± 10% of controls, *p* < 0.05, Fig. 6e). Treatment with strychnine normalizes GAT3 in cerebellar slices of hyperammonemic rats (77 ± 11% of controls, Fig. 6e).

These results show that enhanced glycinergic neurotransmission contributes to neuroinflammation in hyperammonemia. Another aim of this study was to shed light on the mechanisms by which hyperammonemia enhances glycinergic neurotransmission. It has been shown that glycine receptors are modulated by extracellular cGMP at physiological (nM) concentrations, reducing its activation by glycine [12]. Extracellular cGMP levels are reduced in the cerebellum of hyperammonemic rats [13]. We hypothesized that enhanced glycinergic neurotransmission and neuroinflammation in the cerebellum of hyperammonemic rats may be due to the reduced levels of cGMP and that the normalization of their levels could reverse them.

To test this hypothesis, we assessed if the addition of extracellular cGMP to cerebellar slices of hyperammonemic rats normalizes the above mechanisms.

### Extracellular cGMP reduces astrocytes and microglia activation and normalizes membrane expression of ADAM17 and TNFR1 in cerebellar slices of hyperammonemic rats

**Fig. 7:**
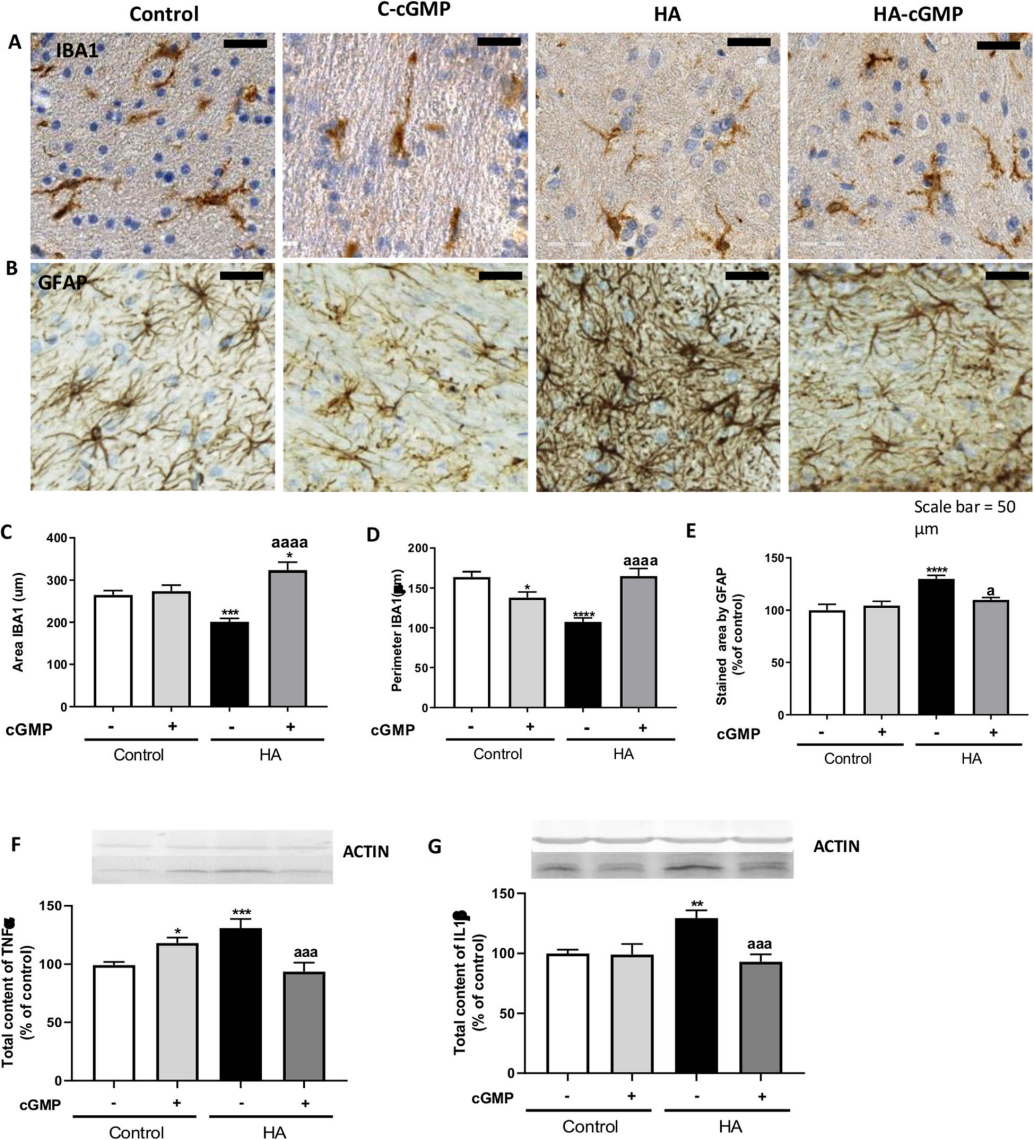
Extracellular cGMP reduces astrocytes and microglia activation and the content of TNFα and IL1β in cerebellum from hyperammonemic (HA) rats. Immunohistochemistry was performed in cerebellar slices from control and hyperammonemic rats treated or not with strychnine as indicated in methods with DAB staining using antibodies against IBA1 (**a**) and GFAP (**b**). To analyze the activation of microglia the area (**c**) and perimeter (**d**) of the cells was measured using the Image J analysis software as described in the “Methods” section. The GFAP stained area was measured as an indicator of astrocytes activation (**e**) as described in the “Methods” section. The content of TNFα (**f**) and IL1β (**g**) was measured by western blot. Values are the mean ± SEM of 6 rats per group in **a-e** and of 30-83 rats per group in **f-g**. Values significantly different from control rats are indicated by asterisk and from hyperammonemic rats are indicated by “a”. *p < 0.05, **p < 0.01, ***p < 0.001, ****p < 0.0001; a *p* < 0.05, aaa *p* < 0.001, aaaa *p* < 0.0001. Data were analyzed using a one-way analysis of variance (ANOVA) followed by Turkey post hoc

We assessed the effects of the addition of extracellular cGMP on the activation of microglia and astrocytes by immunohistochemistry (Fig. 7a and b). Extracellular cGMP normalized the area stained by Iba-1 to 323 ± 18μm^2^ (*p* < 0.0001) (Fig. 7c) and the perimeter of microglia to 165 ± 9 μm (*p* < 0.0001) (Fig. 7d) in hyperammonemic rats. Extracellular cGMP also reduced significantly (*p* < 0.05) the area stained by GFAP in hyperammonemic rats to 109 ± 2% of controls (Fig. 7e). Extracellular cGMP also normalized the content of TNFα and IL1β in cerebellar slices of hyperammonemic rats (Fig. 7f and g, respectively).

**Fig. 8:**
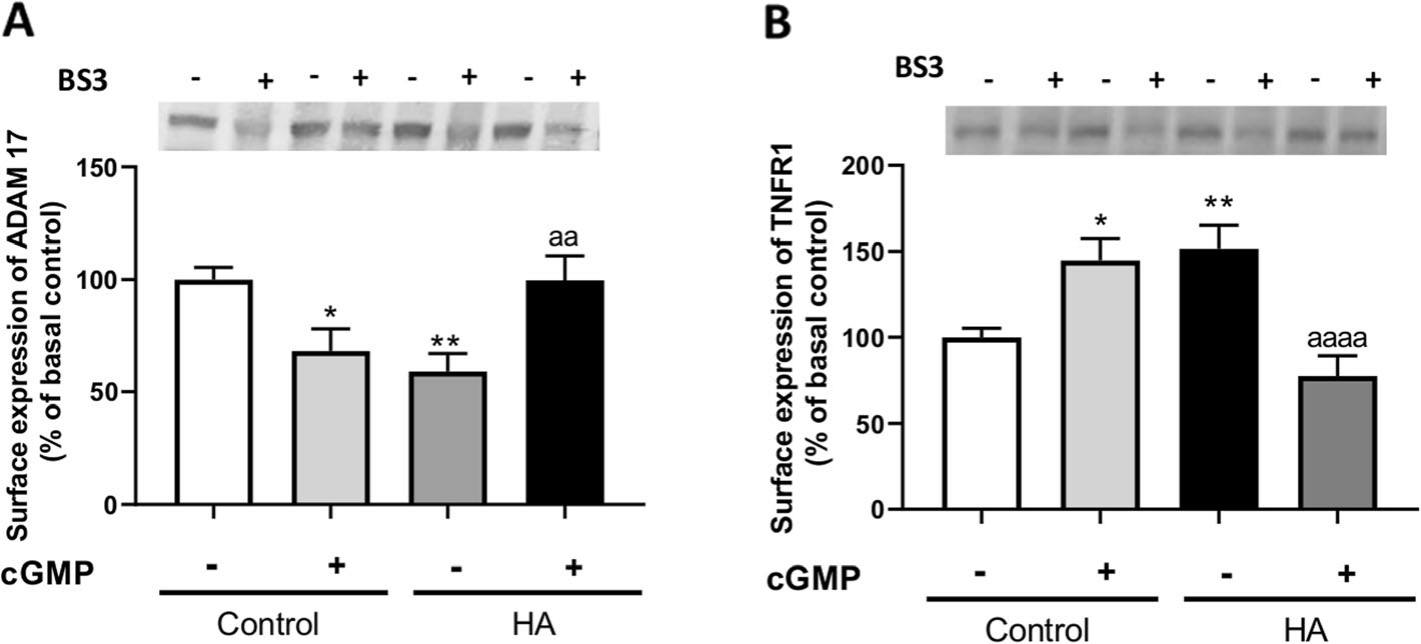
Extracellular cGMP normalizes membrane expression of ADAM17 and TNFR1 in cerebellar slices of hyperammonemic rats. Membrane expression of ADAM17 (**a**) and TNFR1 (**b**) were analyzed using BS3 cross-linker procedure in slices from control and hyperammonemic (HA) rats treated or not with extracellular cGMP as described in the “Methods” section. Values are the mean ± SEM of 20–39 rats per group. Values significantly different from control rats are indicated by asterisk and from hyperammonemic rats are indicated by “a”. *p < 0.05, **p < 0.01; aa *p* < 0.01, aaaa *p* < 0.0001. Data were analyzed using a one-way analysis of variance (ANOVA) followed by Turkey post hoc

Extracellular cGMP, as strychnine completely normalized the membrane expression of ADAM17 to 99 ± 10% (*p* < 0.01, Fig. 8a) and of TNFR1 to 77 ± 11% (*p* < 0.0001, Fig. 8b) of control rats. This indicates that extracellular cGMP could reduce TNFR1 membrane expression by normalizing membrane expression of ADAM17 which would be due to inhibition of the glycine receptor.

### Extracellular cGMP normalizes the nuclear content of NF-κB, glutaminase, mRNA, and protein of TNFα, IL1β, and HMGB1 in Purkinje cells of hyperammonemic rats

**Fig. 9:**
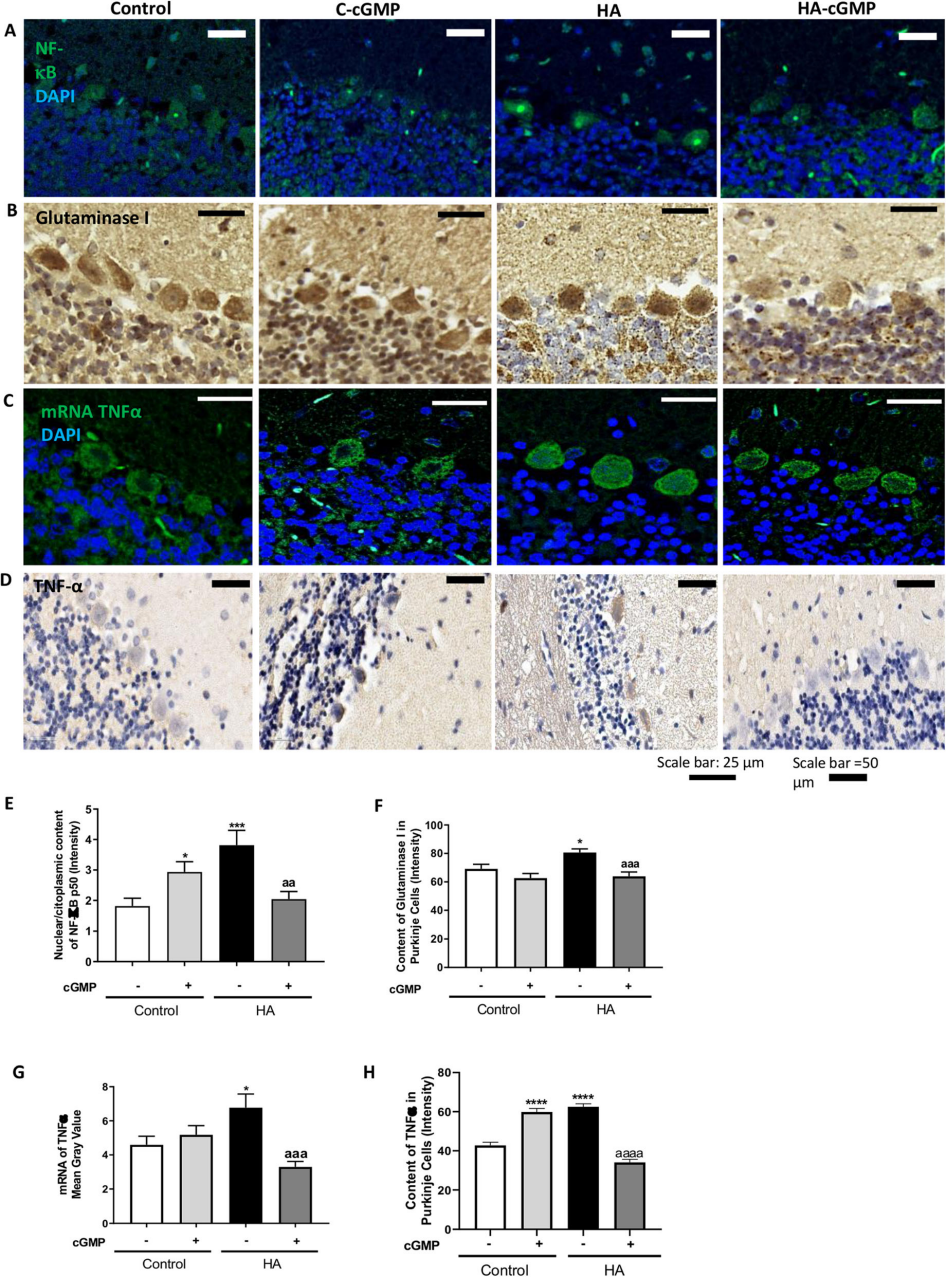
Extracellular cGMP normalizes the nuclear content of NF-κB and the levels of glutaminase, TNFα, and mRNA of TNFα in Purkinje cells of hyperammonemic rats. Immunofluorescence was performed using anti-NF-KB (**a**) and anti-mRNA of TNFα (**c**) as described in the “Methods” section. Immunohistochemistry was performed as indicated in methods with DAB staining using antibodies against glutaminase (**b**) and TNFα (**d**). Representative images are shown. The ratio nuclear/cytoplasmic content of NF-KB (**e**), the content of glutaminase (**f**), mRNA of TNFα (**g**), and TNFα (**h**) was quantified in Purkinje cells as described in the “Methods” section. Values are mean ± SEM of 6 rats per group. Values significantly different from control rats are indicated by asterisk and from hyperammonemic rats are indicated by “a”. *p < 0.05, ***p < 0.001, ****p < 0.0001; aa *p* < 0.01, aaa *p* < 0.001, aaaa *p* < 0.0001. Data were analyzed using the non-parametric Kruskal-Wallis test followed by Dunnett’s post hoc test (in **e**) and a one-way analysis of variance (ANOVA) followed by Turkey post hoc (in **f-h**)

**Fig. 10:**
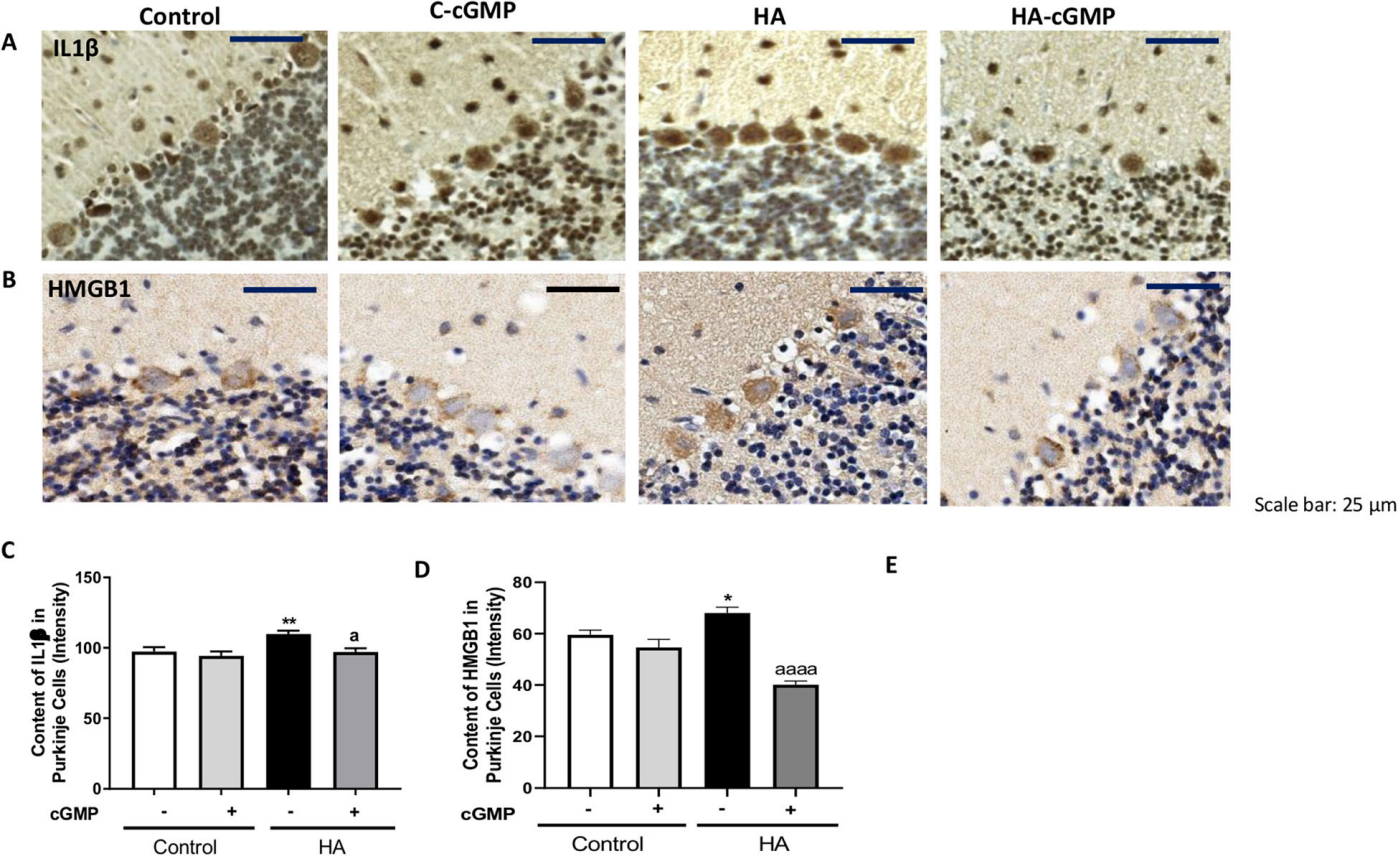
Extracellular cGMP normalizes the IL1β and HMGB1 in Purkinje cells of hyperammonemic rats. Immunohistochemistry was performed as indicated in methods with DAB staining using antibodies against IL1β (**a**) and HMGB1 (**b**). Representative images are shown. The content of IL1β (**c**) and HMGB1 (**d**) was quantified in Purkinje cells as described in the “Methods” section. Values are mean ± SEM of 6 rats per group. Values significantly different from control rats are indicated by asterisk and from hyperammonemic rats are indicated by “a”. *p < 0.05, **p < 0.01; a *p* < 0.05, aaaa *p* < 0.0001. Data were analyzed using a one-way analysis of variance (ANOVA) followed by Turkey post hoc

We then assessed the effect of extracellular cGMP on the TNFR1 downstream pathway as described above. As shown in Fig. 9a and e, extracellular cGMP normalizes the nuclear content of the p50 subunit of NF-κB, glutaminase (Fig. 9b and f), the amount of mRNA for TNFα (Fig. 9c and g), the total content of TNFα (Fig. 9d and h), IL1β (Fig. 10a and c), and HGMB1 (Fig. 10b and d) in Purkinje cells.

This indicates that extracellular cGMP, as strychnine, could be reducing the activation of microglia by a process mediated by the inhibition of glycine receptor and the TNFR1 downstream pathway and by the normalization of HMGB1 in hyperammonemic rats. This supports the idea that enhanced activation of glycine receptor in hyperammonemia would be due to reduced tonic inhibition by extracellular cGMP and may be normalized by increasing it.

## Discussion

**Fig. 11:**
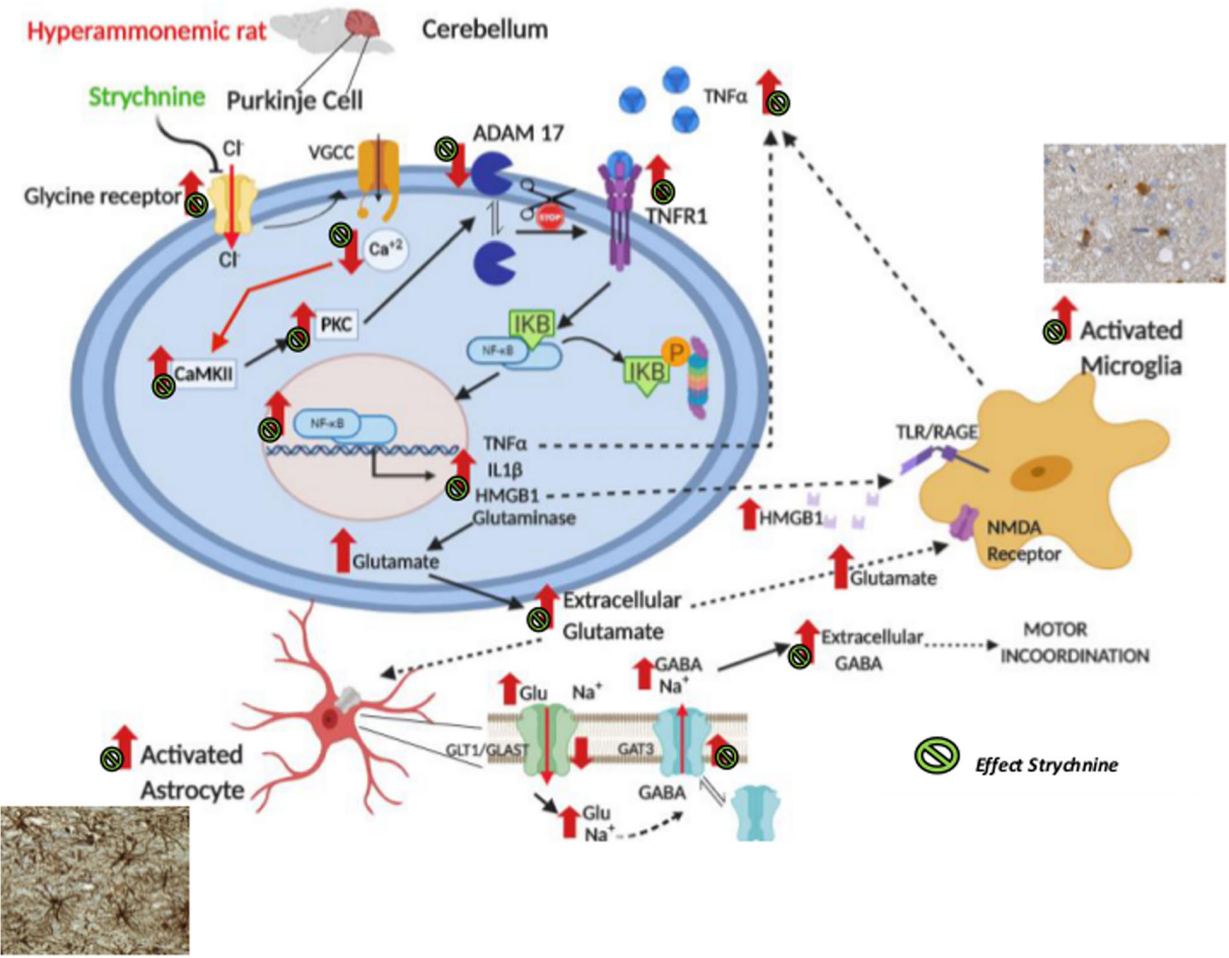
Proposed pathway by which blocking glycine receptors reduces neuroinflammation and restores neurotransmission in cerebellum of hyperammonemic rats through an ADAM17-TNFR1-NF-kB pathway in Purkinje neurons. Glycine receptors are expressed mainly in Purkinje cells. In hyperammonemic rats, enhanced glycinergic neurotransmission leads to reduced membrane expression of ADAM17, resulting in increased surface expression and activation of TNFR1 and of the associated NF-kB pathway. This increases the expression in Purkinje neurons of TNFα, IL-1β, HMGB1, and glutaminase. Increased glutaminase activity leads to increased extracellular glutamate, which increases extracellular GABA. Increased extracellular glutamate and HMGB1 potentiate microglial activation. The effects of hyperammonemia are indicated by red arrows (↑). The effects of strychnine by the green symbol

**Fig. 12:**
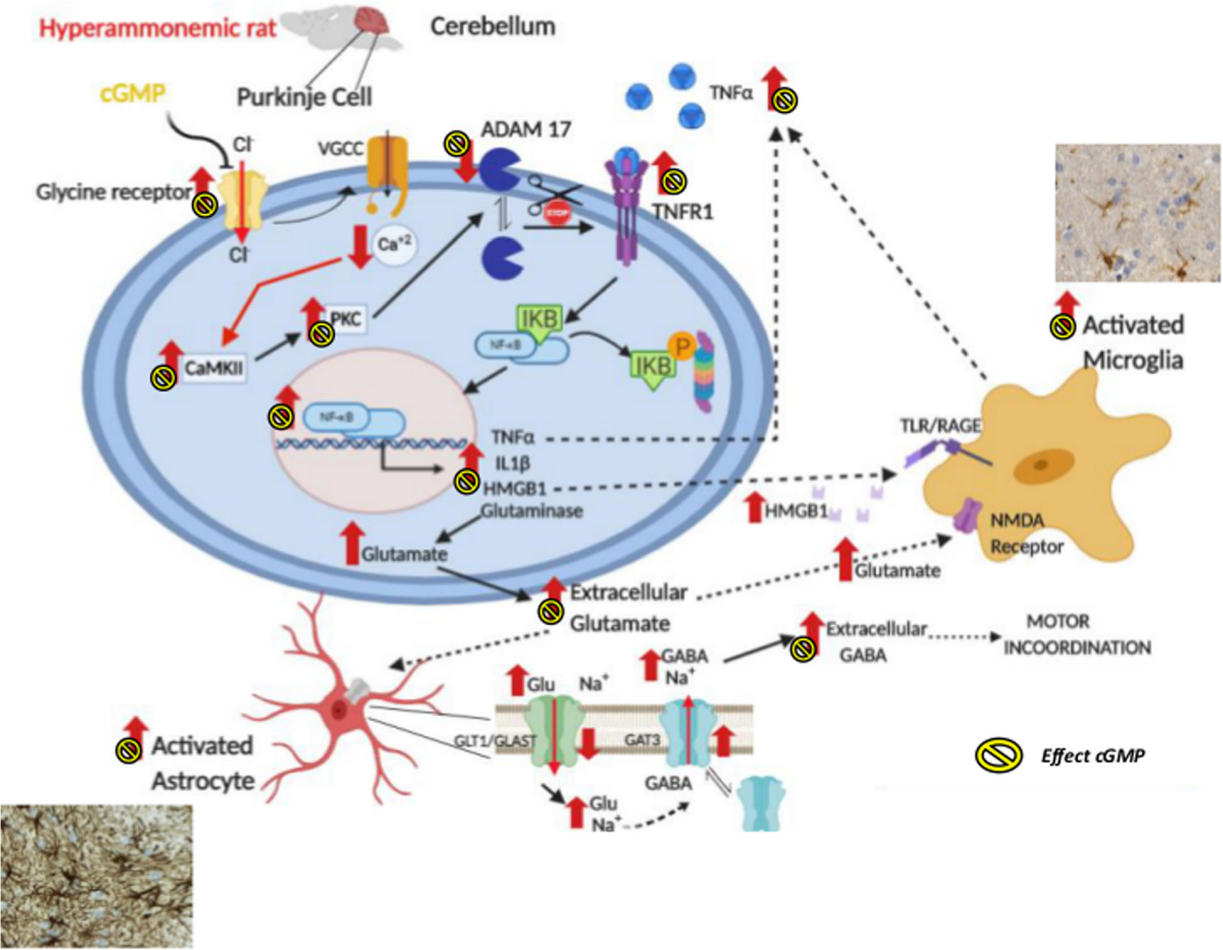
Proposed pathway by which extracellular cGMP through the inhibition of glycine receptors reduces neuroinflammation and restores neurotransmission in the cerebellum of hyperammonemic rats through an ADAM17-TNFR1-NF-kB pathway in Purkinje neurons. The effects of cGMP are the same induced by strychnine, indicating that enhanced glycinergic neurotransmission in hyperammonemia would be due to reduced extracellular cGMP. The effects of hyperammonemia are indicated by red arrows (↑). The effects of cGMP by the yellow symbol

The results reported provide several relevant new contributions and are summarized in Figs. 11 and 12.

A main contribution is the clear demonstration that glycinergic neurotransmission modulates neuroinflammation and activation of microglia and astrocytes. This opens new possibilities to reduce neuroinflammation by acting, for example, on glycine receptors.

This report shows that in the cerebellum of hyperammonemic rats, enhanced glycinergic neurotransmission reduces membrane surface expression of ADAM17, which leads to increased membrane expression and activation of TNFR1 by TNFa. This increases the translocation of NF-kB to the nucleus, leading to enhanced expression of TNFa, IL-1b, HMGB1, and glutaminase in Purkinje neurons. The increased glutaminase activity increases extracellular glutamate, leading to increased extracellular GABA concentration which, in turn, induces motor incoordination. In addition, the increases of neuron-derived HMGB1 and extracellular glutamate contribute to potentiate microglial activation. We discuss below the steps of this pathway and the possible pathological and therapeutic implications of these findings.

We show that strychnine, an antagonist of glycine receptors, reduces neuroinflammation, inducing beneficial effects in cerebellum of hyperammonemic rats. The inhibitory glycinergic neurotransmission in the spinal cord dorsal horn also plays a main role in regulating nociceptive signaling. It has been proposed that targeting glycinergic neurotransmission in the dorsal horn is a good therapeutic strategy to treat specific pathologies such as pain. In chronic pain, this may include approaches to increase glycinergic transmission in spinal pain circuits with agonists and positive modulators of glycine receptors, through inhibition of glycine transporters, gene therapy, and through inhibition of inflammatory mediated pathways [29].

The results support that enhanced activation of glycine receptors in hyperammonemic rats leads to reduced membrane expression of ADAM17, a metalloprotease that is responsible for shedding of TNFR1 [21, 22]. This reduction of ADAM17 would be responsible for the increased TNFR1 in the membrane and activation of the associated pathway.

Immunofluorescence analysis shows that glycine receptors are mainly expressed in Purkinje neurons, with comparatively poor or no expression in microglia and astrocytes. We therefore studied the steps associated to TNFR1 activation in Purkinje neurons.

Chronic hyperammonemia increases NF-kB levels in the nucleus of Purkinje neurons and this is a consequence of increased activation of TNFR1 [26]. It is shown that increased NF-kB in the nucleus increases the content in Purkinje neurons of TNFα, IL-1β, glutaminase, and HMGB1 (Figs. 4 and 5). The increased levels of glutaminase produce more glutamate resulting in increased levels of extracellular glutamate and glutamate uptake by activated astrocytes. In these activated astrocytes, the function of the GABA transporter GAT3 is reversed and instead of taking up GABA, releases GABA to the extracellular fluid, resulting in increased levels of extracellular GABA which, in turn, induce motor incoordination in hyperammonemic rats (Fig. 6).

In the spinal cord dorsal horn neurons, it has also been shown that synaptic inhibition mediated by glycinergic and GABAergic receptors on excitatory neurons is modulated by inflammation associated to pain, through a change in the functioning of existing glycine and GABA_A_ receptors [30].

There are at least two mechanisms by which these changes in Purkinje neurons are transmitted to microglia to keep them in an activated form. One mediator synthesized in Purkinje neurons which is released and activates microglia is HMGB1 and another is glutamate itself (Figs. 5d and 6a, respectively). We discuss below in some detail the different steps of this general process.

The results reported also show that all this process summarized in Fig. 11 is a consequence of enhanced activation of glycine receptors in Purkinje neurons of hyperammonemic rats, as demonstrated by the fact that blocking glycine receptors with strychnine completely prevents all the process (Fig. 11).

Moreover, it is also shown that the enhanced glycinergic neurotransmission in the cerebellum of hyperammonemic rats would be a consequence of the lower levels of extracellular cGMP, which physiologically inhibits tonically glycine receptors. We show that increasing extracellular cGMP in cerebellar slices of hyperammonemic rats prevents all the processes summarized in Fig. 12 exactly as strychnine (Fig. 11), thus supporting that the lower levels of extracellular cGMP in hyperammonemia lead to reduced inhibition of the receptors, resulting in increased glycinergic neurotransmission which, in turn, induces neuroinflammation.

Some details of the above general process are discussed below.

A main step in the process summarized in Fig. 11 is the increased membrane expression and activation of TNFR1. It has been already shown that increased membrane expression of TNFR1 mediates the induction of neuroinflammation and motor incoordination in the cerebellum of hyperammonemic rats [3]. It is now shown that increased membrane expression of TNFR1 in hyperammonemia would be due to enhanced glycinergic neurotransmission and reduced membrane expression of ADAM17. TNFR1 in membrane may be modulated by shedding mediated by ADAM17 [21–23]. Hyperammonemia reduces ADAM17 in the membrane, reducing TNFR1 shedding and resulting in increased TNFR1 in the membrane. Hyperammonemia reduces ADAM17 in the membrane by increasing PKC activity, as supported by the fact that inhibition of PKC normalizes the levels in the membrane of both ADAM17 and TNFR1.

The mechanism by which hyperammonemia increases PKC activity in the cerebellum has been already described and is mediated by increased activity of CaMKII which in turn, indirectly, enhances PKC activity [2]. Increased activity of CaMKII in hyperammonemia is due to increased phosphorylation at the residue Thr286, which is biphasically modulated by calcium concentration [31]. In hyperammonemia basal levels of calcium in Purkinje neurons are reduced resulting in increased phosphorylation and activity of CaMKII [13]. Blocking glycine receptors in hyperammonemic rats induces a voltage-dependent calcium-channels-mediated increase of calcium in Purkinje neurons that reduces CaMKII phosphorylation and activity [13], thus reducing PKC activity (Fig. 11).

A main mechanism by which PKC modulates membrane expression of ADAM17 is mediated by phosphorylation in threonine 735 through the PKC-ERK/p38 pathway [24, 25]. However, the results reported here show that this mechanism is not responsible for the effects of hyperammonemia on ADAM17. Another mechanism by which the membrane expression of ADAM17 can be reduced by PKC is by enhancing its release in exosomes. This has been shown in cells stimulated with phorbol-12-myristate-13-acetate (PMA), a PKC activator, or lipopolysaccharide (LPS) [32]. It is possible that a similar mechanism would contribute to the reduced levels of ADAM17 in the membrane in the cerebellum of hyperammonemic rats.

Blocking glycine receptors in hyperammonemic rats reduces CaMKII phosphorylation and activity [13], thus reducing PKC activity and normalizing ADAM17 and TNFR1 levels in the membrane (Fig. 3).

In rats with chronic hyperammonemia increased membrane expression of TNFR1 leads to increased nuclear translocation of NF-κB and, as a consequence, an increase of TNFα, IL1β, and glutaminase expression [3, 26]. We show here that, in addition, this pathway also increases the levels of HMGB1 in Purkinje neurons (Fig. 5b and d). All these steps are also reversed by blocking glycine receptors with strychnine, indicating that they are also a consequence of enhanced glycinergic neurotransmission in hyperammonemia (Fig. 11).

The increase of glutaminase results in increased formation of glutamate. An increase in TNFa-induced formation of glutamate through increased glutaminase expression has been reported in several pathological situations [33–36]. TNFa-induced increase of glutamate has been proposed as the central mechanism by which excessive TNFa harms cerebral function and structure across the range of neurodegenerative diseases including Parkinson’s, Huntington’s and Alzheimer diseases, amyotrophic lateral sclerosis or in septic encephalopathy [37]. TNFa-induced glutamate increase has been also proposed as the most logical therapeutic target in these situations [37]. TNFa-induced increase of glutaminase expression and glutamate levels occur both in microglia [38, 39] and in neurons [36, 40]. The results reported here show that in cerebellum of hyperammonemic rats there is also a TNFa-induced increase of glutaminase expression in Purkinje neurons and of extracellular glutamate. A similar increase has been reported in microglia in cerebellum of hyperammonemic rats [3]. However, glycinergic neurotransmission would modulate this pathway mainly in neurons because glycine receptor is strongly expressed in Purkinje neurons but not in microglia. The inhibition of this pathway in Purkinje cells of hyperammonemic rats by strychnine supports this idea.

In some of the above pathological situations in which TNFa levels and extracellular glutamate reach high levels, this pathway may lead to excitotoxicity and neuronal degeneration and the associated cognitive impairment [37]. In the model of chronic hyperammonemia used here, the increase of TNFa and extracellular glutamate in the cerebellum seems to be milder and does not reach levels high enough to induce neuronal death. However, they are enough to alter neurotransmission, cognitive function, and motor coordination.

The excess of glutamate in the extracellular space must be removed to protect from glutamate excitotoxicity and keep normal synaptic transmission. Extracellular glutamate is removed mainly by astrocytes through the glutamate transporters GLAST and GLT-1 [41–45]. In cerebellum of hyperammonemic rats, the increased levels of extracellular glutamate would result in increased transport through glutamate transporters in activated astrocytes, thus increasing intracellular Na^+^ which would reverse the function of the GABA transporter GAT-3, leading to increased extracellular GABA [3, 9]. This is supported by the fact that blocking the glutamate transporters GLT-1 and GLAST in microdialysis experiments in vivo reduced extracellular GABA in the cerebellum of hyperammonemic rats [3]. In agreement with this, it has been shown that in activated astrocytes the function of the GAT-3 transporter of GABA is reversed, and instead of taking up GABA into the astrocyte, releases GABA, thus increasing extracellular GABA levels [46, 47].

Blocking glycine receptors with strychnine prevents all the pathways in hyperammonemic rats, normalizing TNFa-induced expression of glutaminase and extracellular glutamate levels. This is associated with the normalization of astrocytes activation, expression of GAT-3, and extracellular levels of GABA (Fig. 11).

To confirm that blocking glycine receptors normalizes extracellular glutamate and GABA in the cerebellum of hyperammonemic rats in vivo, we analyzed these neurotransmitters by microdialysis in the cerebellum of freely moving rats. Both extracellular glutamate and GABA concentrations were increased in hyperammonemic rats. Blocking glycine receptors by the administration of strychnine through the microdialysis probe reduced extracellular glutamate and GABA to normal levels, thus supporting that the above process (Fig. 11) is also occurring in the cerebellum in vivo.

Similar effects are obtained when the activation of glycine receptor is reduced by adding extracellular cGMP (Fig. 12). Normalization of extracellular GABA levels is associated with the restoration of motor coordination in hyperammonemic rats [3, 6].

The activation of the TNFR1-NF-kB pathway in Purkinje neurons of hyperammonemic rats not only alters neurotransmission but also promotes neuroinflammation and microglial activation. Increased nuclear NF-kB also enhances transcription of IL-1b, TNFa, and HMGB1 in Purkinje neurons. IL-1b and TNFa are pro-inflammatory cytokines that promote neuroinflammation by activating their receptors in different cell types, including microglia.

HMGB1 is released from neurons in situations associated with neuroinflammation such as ischemic injury, traumatic injury, intracerebral hemorrhage, and spinal cord injury [48]. HMGB1 released by neurons leads to activation of microglia by binding to the TLR4 receptor [27, 28, 49]. In hyperammonemic rats, the release of HMGB1 from Purkinje neurons would contribute to keep activation of microglia by a similar mechanism. Moreover, strychnine would be reducing the activation of microglia by reducing HMGB1 expression and release in Purkinje neurons.

Another mediator released by Purkinje neurons in hyperammonemic rats that would contribute to microglia activation is glutamate itself. Microglia expresses most types of glutamate receptors, including NMDA receptors. Activation of NMDA receptors by glutamate in microglia leads to morphological activation and release of inflammatory mediators that promote microglial polarization [50, 51]. Glutamate released from Purkinje neurons in hyperammonemic rats would also contribute to keep microglial activation. The reduction of this glutamate release by strychnine would contribute to reduced activation of microglia.

This report also shows that the addition of extracellular cGMP, which does not cross the cell membrane, also prevents all the processes triggered by enhanced activation of glycine receptors in Purkinje neurons of hyperammonemic rats (Fig. 12). Extracellular cGMP restores membrane expression of ADAM17 and TNFR1, normalizes nuclear expression of NF-kB and expression of glutaminase, IL-1b, TNFa, and HMGB1 in Purkinje neurons and activation of microglia and astrocytes. It has been already shown that extracellular cGMP also normalizes GAT-3 and extracellular levels of glutamate and GABA in the cerebellum of hyperammonemic rats and restores motor coordination [3].

These results support the idea that increased glycinergic neurotransmission in hyperammonemic rats [10] would be a consequence of reduced inhibition of the receptor by extracellular cGMP, which concentration is reduced in cerebellum of hyperammonemic rats [13–15, 52].

It has been repeatedly shown that increasing cGMP levels by using inhibitors of the phosphodiesterases (PDEs) that degrades it (mainly PDE5) prevents or reverses neuroinflammation in models of different pathologies including hepatic encephalopathy, hyperammonemia, focal ischemia, multiple sclerosis, Huntington’s, or Alzheimer’s diseases [53–59]. Increasing only extracellular cGMP also reverses neuroinflammation in models of chronic hyperammonemia and hepatic encephalopathy [3, 60]. This report also identifies a mechanism by which these protective effects could be occurring, by reducing increased activation of glycine receptors and the associated activation of the TNFR1-NF-kB pathway.

## Conclusions

In summary, we show that glycinergic neurotransmission modulates neuroinflammation. In hyperammonemic rats, enhanced glycinergic neurotransmission leads to reduced membrane expression of ADAM17, resulting in increased surface expression and activation of TNFR1 and of the associated NF-kB pathway. This leads to increased expression in Purkinje neurons of TNFa, IL-1b, HMGB1, and glutaminase. Increased glutaminase activity leads to increased extracellular levels of glutamate, which leads to increased extracellular levels of GABA. This altered neurotransmission leads to motor incoordination. Increased extracellular glutamate and HMGB1 potentiate microglial activation by acting on TLR4 and NMDA receptors, respectively.

Blocking glycine receptors with strychnine or extracellular cGMP completely prevents the above pathway in hyperammonemic rats. This indicates that enhanced glycinergic neurotransmission in hyperammonemia would be due to reduced extracellular levels of cGMP. These results shed some light on possible new therapeutic target pathways for pathologies associated to neuroinflammation.

## Abbreviations

BS3: Bis(sulfosuccinimidyl) suberate
TNFa: Tumor necrosis factor alpha
TNFR1: TNFα receptor 1
NF-κB: Nuclear factor kappa B
GAT3: GABA transporter type 3
GABA: γ-aminobutyric acid
cGMP: Cyclic guanosine monophosphate
IL-1β: Interleukin 1 beta
ADAM17: ADAM metallopeptidase domain 17
GLAST: Glutamate aspartate transporter 1
GLT1: Glutamate transporter 1
HMGB1: High mobility group box 1 protein
Iba1: Ionized calcium-binding adapter molecule 1
GFAP: Glial fibrillary acidic protein
DAPI: 4′,6-diamidino-2-phenylindole
HPLC: High-performance liquid chromatography
ANOVA: One-way analysis of variance
